# Characterization of the complete plastome of *Alopecurus aequalis* (Poaceae), a widespread weed

**DOI:** 10.1080/23802359.2019.1693925

**Published:** 2019-11-22

**Authors:** Rong Wang, Qing-Jun Wang, Xiao-Jian Qu, Shou-Jin Fan

**Affiliations:** Key Lab of Plant Stress Research, College of Life Sciences, Shandong Normal University, Ji’nan, Shandong, China

**Keywords:** *Alopecurus aequalis*, plastome, phylogenomics

## Abstract

*Alopecurus aequalis* is a predominant weed species that distributes widely in North temperate regions. The complete plastome of *A. aequalis* is reported here. It is a circular molecular of 136,382 bp in length and consists of a large single-copy region (LSC: 80,455 bp), a small single-copy region (SSC: 12,849 bp), and two inverted repeats regions (IRs: 21,539 bp). GC content is 38.3%. This plastome encodes 112 unique genes, including 78 protein-coding genes, 30 tRNAs, and 4 rRNAs. Phylogenetic tree shows that *A. aequalis* is sister to *Poa annua*.

*Alopecurus aequalis* (shortawn foxtail) belongs to Pooideae of Poaceae. It is a widespread annual weed growing on irrigation ditches, rice fields, damp grasslands and other wet weedy places below 3500 m (Guo et al. 2015). This species is mainly distributed in North temperate region of the world, especially in Europe, temperate Asia and North America. It is closely related to *Beckmannia* and *Poa*, and these three genera are classified into subtribe Poinae (Soreng et al. 2015). As a troublesome weed, it can germinate under many different environmental conditions, and has a bad influence on some overwintering crops such as wheat, canola, rice and many early spring vegetables (Morishima and Oka 1980; Wang and Sheng 2007; Zhao et al. 2018). Chemical herbicides are important ways to control this weed, and many studies concentrate on resistance of *A. aequalis* to herbicides such as mesosulfuron-methyl (Huang et al. 2010; Zhao et al. 2017; Guo et al. 2018). A better understanding of the germination ecology of this weed can provide the basis for more control measures (Zhao et al. 2018). Besides, many species of *Alopecurus* are fine pasture, such as *Alopecurus pratensis* (Shutt et al. 1928), *Alopecurus arundinaceus* (Gudleifsson et al. 1986) and *Alopecurus brachystachyus* (Holzworth 2006).

Silica-dried leaves of *A. aequalis* were collected from Chengde, Hebei, China (117°29′E, 42°4′N). Voucher specimen (No.22) was stored at College of Life Science, Shandong Normal University. Total genome DNA was isolated using a modified CTAB-based protocol (Wang et al. 2013). DNA library preparation and sequencing were conducted by Illumina MiSeq platform at Novogene (Beijing, China). After obtaining sequencing data, we used Organelle Genome Assembler (OGA, https://github.com/quxiaojian/OGA) to assemble plastome (Qu 2019). Annotation was accomplished with Plastid Genome Annotator (PGA, https://github.com/quxiaojian/PGA) (Qu et al. 2019). We used Geneious v9.1.4 to do manual correction. All 78 protein-coding genes were selected to construct the maximum likelihood (ML) tree by RAxML v8.2.10 (Alexandros 2014), using 1000 bootstrap replicates with GTRCAT model after alignment using MAFFT v7.313 (Kazutaka and Standley 2013).

The complete plastome of *A. aequalis* (GenBank accession number: MN422306) is a circular molecular of 136,382 bp in length, consisting of two single-copy regions separated by a pair of inverted repeats (IRs) of 21,539 bp. The large single-copy region and the small single-copy region are 80,455 and 12,849 bp, respectively. GC content is 38.3%. This plastome encodes 112 unique genes, among which 78 protein-coding genes, 30 tRNAs, and 4 rRNAs. Phylogenetic tree shows that *A. aequalis* is sister to *Poa annua* (Figure 1).

**Figure 1.**
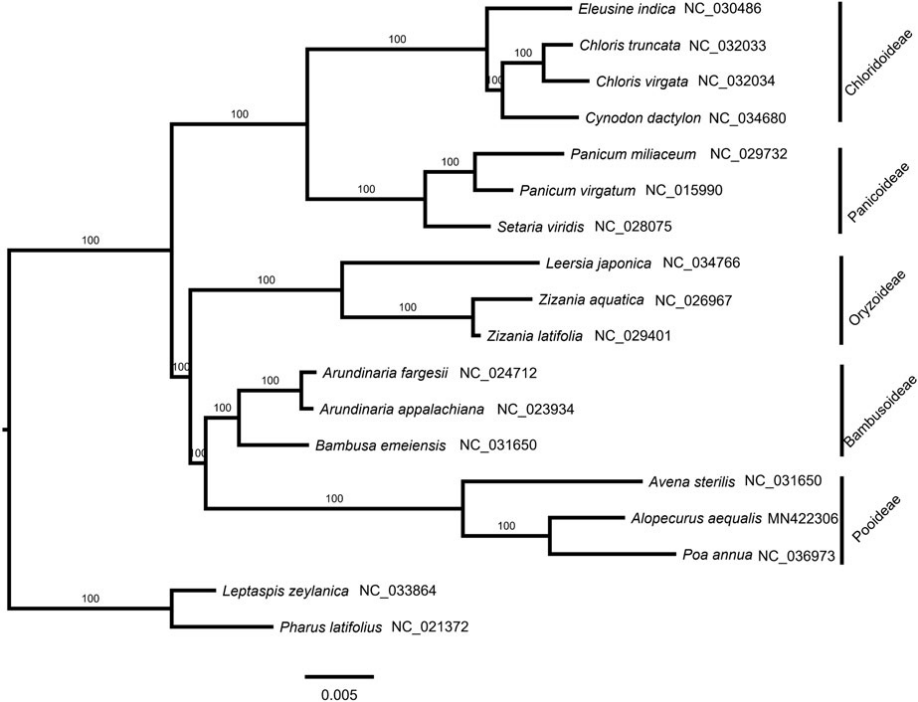
A maximum-likelihood (ML) tree inferred from 78 plastome genes is shown. *Leptaspis zeylanica* and *Pharus latifolius* from Poaceae are used as outgroup. The numbers on branches are bootstrap support values.
